# Blood smears examination and prevalence of malaria in Addis Zemen Town, Northwest Ethiopia (2013–2021): a retrospective study

**DOI:** 10.1186/s40794-024-00219-y

**Published:** 2024-05-15

**Authors:** Tilahun Adugna, Lamesgin Zelalem, Gedafaw Alelign

**Affiliations:** Debre Tabor University, 272 Debre Tabor, Ethiopia

**Keywords:** Ethiopia, Addis Zemen, *Plasmodium Falciparum*, Sheni River, Irrigation

## Abstract

**Introduction:**

In Ethiopia, malaria is one of the major public health and socioeconomic problems, though tremendous efforts have been made. Currently, the country has a plan to eliminate malaria by 2030. To achieve this plan, epidemiological studies associated with malaria prevalence with gender, age groups, species types, and seasons are essential. Therefore, the aim of this study was to assess the prevalence of malaria from 2013 to 2021 in Addis Zemen town, Northwest Ethiopia.

**Methods:**

A retrospective study was conducted at assess the trend of malaria prevalence over the last nine years using recorded blood smear reports in the laboratory logbook from governmental health institutions. Trends in malaria cases and the proportion of genders, age groups, species, and seasons over time were compared. The data were analyzed using the SPSS-23 software package.

**Results:**

The overall malaria prevalence between 2013 and 2021 was 10.4%. From all confirmed cases, the minimum and maximum prevalence of malaria cases were recorded in 2018 (2%) and 2016 (33.2%) years, respectively. The infectious rate of males (59.3%) was significantly higher than that of females (40.7%) (*p* < 0.0001). In all survey periods, all age groups were infected by malaria parasites; the majority of the cases were between 15 and 45 years (57%) older than others. Statistically, a greater proportion of *P. falciparum* (80.1%) was recorded than *P. vivax* (18.5%) (*p* < 0.0001). Malaria cases were occurring throughout each month. The relative highest peaks of total malaria cases were observed during the months of September, October, and November. Seasonally, the highest infection rate was observed during spring (40.20%) compared to other seasons.

**Conclusions:**

In conclusion, the study revealed that malaria transmission remained high, which affected males more than females and potentially reproductive ages. Two of the most important *Plasmodium* species were identified and found during all reviewed months and years, though *P. falciparum* was the most prevalent. Hence, the problem can be alleviated by using season-based long-lasting insecticide treated nets, regularly overseeing ongoing irrigation activity, overseeing the reduction of the water level of the Sheni River, health education, and providing immediate patient treatment.

## Introduction

Malaria is a complex disease caused by protozoan parasites belonging to the genus *Plasmodium* that mosquitoes transmit through blood feeding on human hosts [1–3]. Of the five *Plasmodium* species, only *Plasmodium falciparum and Plasmodium vivax* have worldwide distribution [4] and are the predominant species in sub-Saharan Africa (SSA) [5].

Malaria is also one of the most important vector-borne diseases that cause morbidity and mortality throughout the world [6, 7] and one of the major diseases of people living in poverty within developing countries [3, 8]. Although tremendous efforts have been made, it continues to pose a serious challenge to countries in the SSA. Based on the WHO report, there were an estimated 241 million malaria cases in 2020, compared to 227 million cases in 2019. About 95% of all malaria cases were in the WHO African Region (Angola, Burkina Faso, Kenya, and Ethiopia). Over 20-year periods (from 2000 to 2020), the population at risk of malaria in SSA nearly doubled because of the presence of antimalarial drug resistance, mosquito resistance to insecticides, and invasive vector species (*Anopheles stephensi*) [9]. In 2020, there were an estimated 627,000 malaria deaths worldwide compared to 558,000 deaths in 2019. About 47,000 additional malaria deaths were due to disruptions in the provision of malaria prevention, diagnosis, and treatment during the pandemic [9].

In Ethiopia, malaria is the leading health problem because three-fourths (75%) of the total area of the country is malarious, and more than two-thirds (approximately 68%) of the total population lives below 2,000 m below sea level [10, 11]. In the country, the nature of malaria transmission is seasonal and unstable, with major transmission occurring from September to mid-December, following the main rainy season (June-August), and minor transmission occurring during March-May [12–18]. Besides, it varies with elevations, temperatures and rainfall [19–21].

Recently, in Ethiopia, malaria morbidity and mortality rates have shown a significant reduction over time. The number of confirmed malaria cases and death rate decreased by 47% and 58%, respectively, between 2016 and 2019 [5]. These results are obtained due to early diagnosis, quick treatment of cases, prevention and control of malaria among pregnant women using intermittent preventive therapy, and vector control methods including long-lasting insecticidal nets (LLINs) and indoor residual sprayings (IRSs) [5, 21]. However, retrospective data shows that the overall trend of malaria prevalence in many SSA-countries like Ethiopia is inadequate [3, 5]. In particular, this is true in Addis Zemen town; the overall trend of malaria prevalence has not studied so far [22], except for the species composition and ecology of anophelines [23]. Epidemiological data from known health facilities laboratory registration books over long periods of time is vital to designing timely and appropriate interventions. That is, generating evidence-based data on prevalence and the distribution of malaria over sex, age, and season from the registration books in the years is so essential. Such information is essential to evaluating the impact of previous intervention strategies as well as to designing evidence-based interventions. Hence, this study aimed to assess the prevalence of malaria in the past nine years at two health facilities in Addis Zemen Town, Northwest Ethiopia.

## Methods

### Study area description

**Fig. 1:**
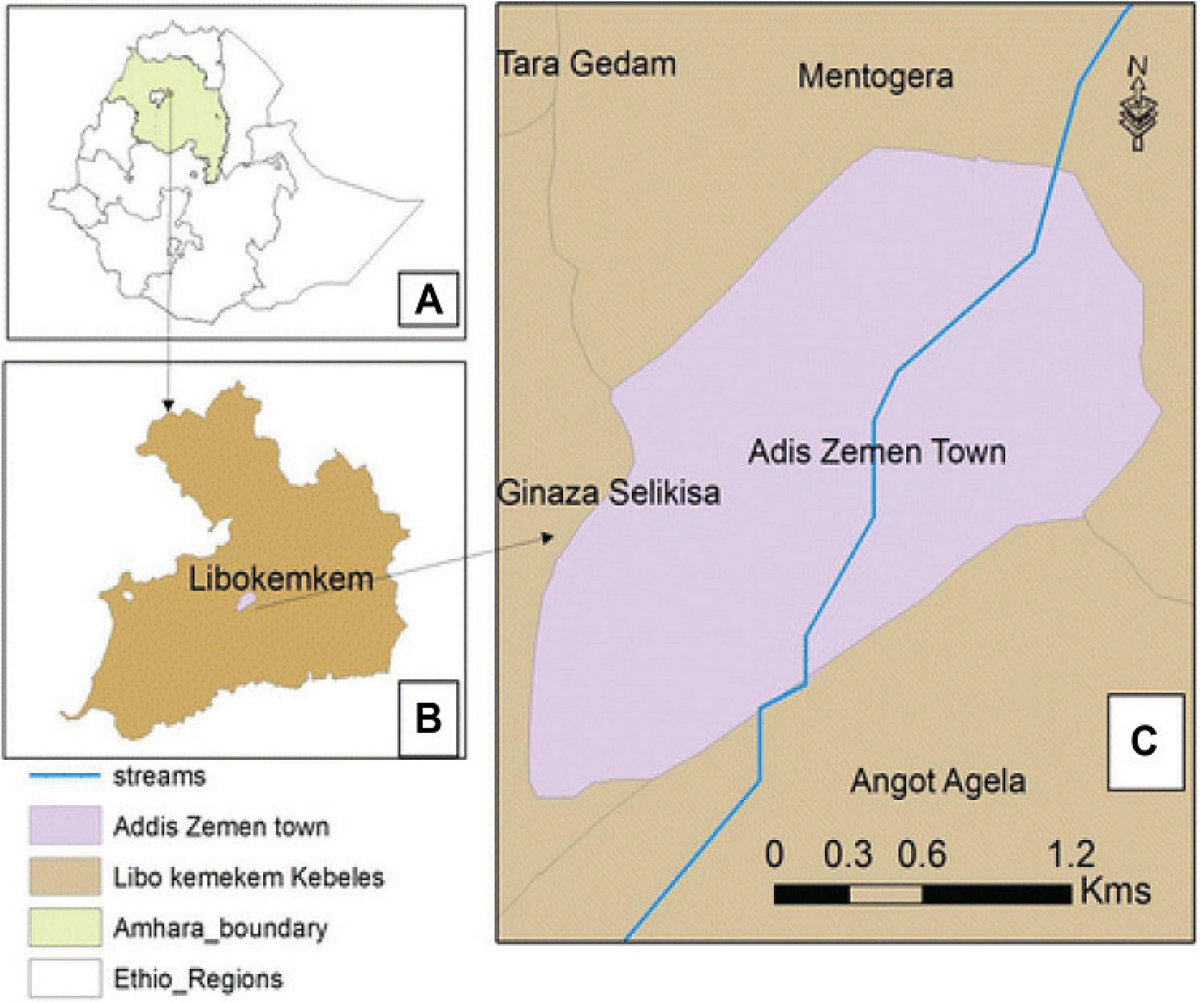
Map of study area: **(a)** Ethiopia, **(b)** Libo-Kemekem district, **(c)** Addis Zemen town, **(d)** Sheni river [22]

This study was conducted in Addis Zemen town, South Gondar, Amhara region, northwestern Ethiopia. Addis Zemen town is the capital of Libo-Kemkem district (Fig. 1). The district is situated at 11° 54′ 36” N and 37° 15′ 36” E with an elevation of 2,000 m above sea level. This town is located 656 km away from Addis Ababa (the capital city of the Ethiopia), 76 km away from Bahir Dar (the capital city of the Amhara region), and 61 km away from Debre Tabor town (the capital city of North Gondar administrative zone). According to the Addis Zemen Town Administration Office (Unpub. Report, 2021), the total population of the town was 40,798 (male = 19,601 and female = 21,197).

Addis Zemen town covers about 16.86 km^2^, which is divided into 4-kebeles (the smallest administrative unit). The town receives a unimodal rainfall of approximately 1,300 mm per year, mostly between June and August. The mean annual temperature is 19.7 °C. In the town, there was about 5-health facilities (2-government, 1-hospital and 1-health center; and 3-private medium clinics). Besides, there were 2 drugstores. In the town, there is the Sheni River, which is used for irrigation, swimming, washing clothes, and extracting sand for town residents [22]. Previous investigations have shown that the town of Addis Zemen has been considered a malaria spot area because large numbers of malaria cases have been recorded in the years [24, 25]. In this town, one of the carriers responsible for malaria in Ethiopia has been identified, *Anopheles gambiae* s.l [23].

### Study design and population

A retrospective study was conducted to determine the nine (from 2013 to 2021) years of malaria prevalence from governmental health institutions (Addis Zemen Hospital and Health Center) found in Addis Zemen Town. The town has been divided into four ‘kebeles’. The study population included only residents of the four ‘Kebeles’ (the smallest administrative unit) in the town who had visited the health institutions during the study period and were only suspected and diagnosed with malaria. Therefore, this study was unable to discuss asymptomatic individuals, and this can be considered a limitation of this study.

### Data collection

Nine-year (2013–2021) retrospective data on malaria prevalence’s were collected from the blood film malaria registration laboratory logbooks at Addis Zemen hospital and Addis Zemen health center, Addis Zemen town, Amhara Region, Northwestern Ethiopia. In these health facilities, peripheral smear examination of a well-prepared and well-stained blood film has been used as the gold standard for confirming the presence of the malaria parasite as per the WHO protocol [26]. In Ethiopia, the staining techniques and blood film examinations for malaria parasite detection are conducted according to a standard operating procedure in each hospital and health center throughout the country [13].

### Data quality control

The retrospective data was taken from the primary record books of the Addis Zemen hospital and health center. The information about every member of the community was registered during their visit to the above health institutions. All necessary data were collected independently by four well trained health workers and cross-checked with each other, and finally confirmed by all authors. Moreover, external quality assurance was done by Amhara Regional Health Bauru. All incomplete data were excluded.

### Data analysis

The data was entered in Microsoft Excel data sheets, cross-checked, and analyzed using the SPSS-23 software package. Descriptive statistics were employed to calculate frequencies and percentages of overall malaria prevalence, trends of malaria transmission in terms of seasons, years, sex, age groups, and species of malaria parasite. Tables and figures were used to describe the analyzed data (sex, age, and season, *Plasmodium* species). For the chi-square test, a *p*-value < 0.05 was taken as statistically significant.

## Results

### Annual malaria case trends

During the past nine years, from January 2013 to December 2021, a total of 44,289 malaria suspected cases were examined using a microscope in Addis Zemen Hospital and Addis Zemen Health Center. Of these, 4,626 (10.4%) were positive for malaria in both health institutions. The number of suspected malaria cases gradually increased from 2013 to 2016 and then decreased until 2018. It then began to rise, peak in 2020, and then gradually decline (Table 1). Similarly, the number of positive malaria cases showed a gradual increment from 2013 (3.9%, 181/4626) to 2016 (33.2%, 1534/4626) and then decreased until 2018 (2%, 94/4626). It then began to rise, and a peak in 2020 (20.4%, 942/4626), and then gradually declined. From all confirmed cases, the minimum and the maximum prevalence of malaria cases were recorded in 2018 (2%, 94/4626), and 2016 (33.2%, 1534/4626) years, respectively (Table 1).

**Table 1:** Slide positivity rates and species composition of malaria parasite in Addis Zemen hospital and health center, from 2013–2021

Years	Total Tested cases	Total Positive Cases				Malaria species (%)		
		Total (%)	P.f (%)	P.v (%)	Mixed (%)	P.f (%)	P.v (%)	Mixed (%)
2013	2895	181(6.3)	116(4)	54(1.9)	11(0.4)	116(64.1)	54(29.8)	11(6.1)
2014	3144	232(7.3)	118(3.8)	107(3.4)	7(0.2)	118(50.9)	107(46.1)	7(3)
2015	5216	1016(19.5)	722(13.8)	285(5.5)	9(0.2)	722(71.1)	285(28.1)	9(0.9)
2016	9374	1534(16.4)	1297(13.8)	222(2.4)	15(0.2)	1297(84.6)	222(14.5)	15(1)
2017	4482	153(3.4)	118(2.6)	35(0.8)	0(0)	118(77.1)	35(22.9)	0(0)
2018	3153	94(3)	65(2.1)	27(0.9)	2(0.1)	65(69.1)	27(28.7)	2(2.1)
2019	4998	289(5.8)	262(5.2)	23(0.5)	4(0.1)	262(90.7)	23(8.0)	4(1.3)
2020	8865	942(10.6)	864(9.8)	67(0.8)	11(0.1)	864(91.7)	67(7.1)	11(1.2)
2021	2162	185(8.6)	143(6.6)	38(1.8)	4 (0.2)	143(77.3)	38(20.5)	4 (2.2)
Total	44,289	4,626(10.4)	3,705(8.4)	858(1.9)	63(0.1)	3705(80.1)	858(18.5)	63(1.3)

### Malaria incidence rate by sexes

**Fig. 2:**
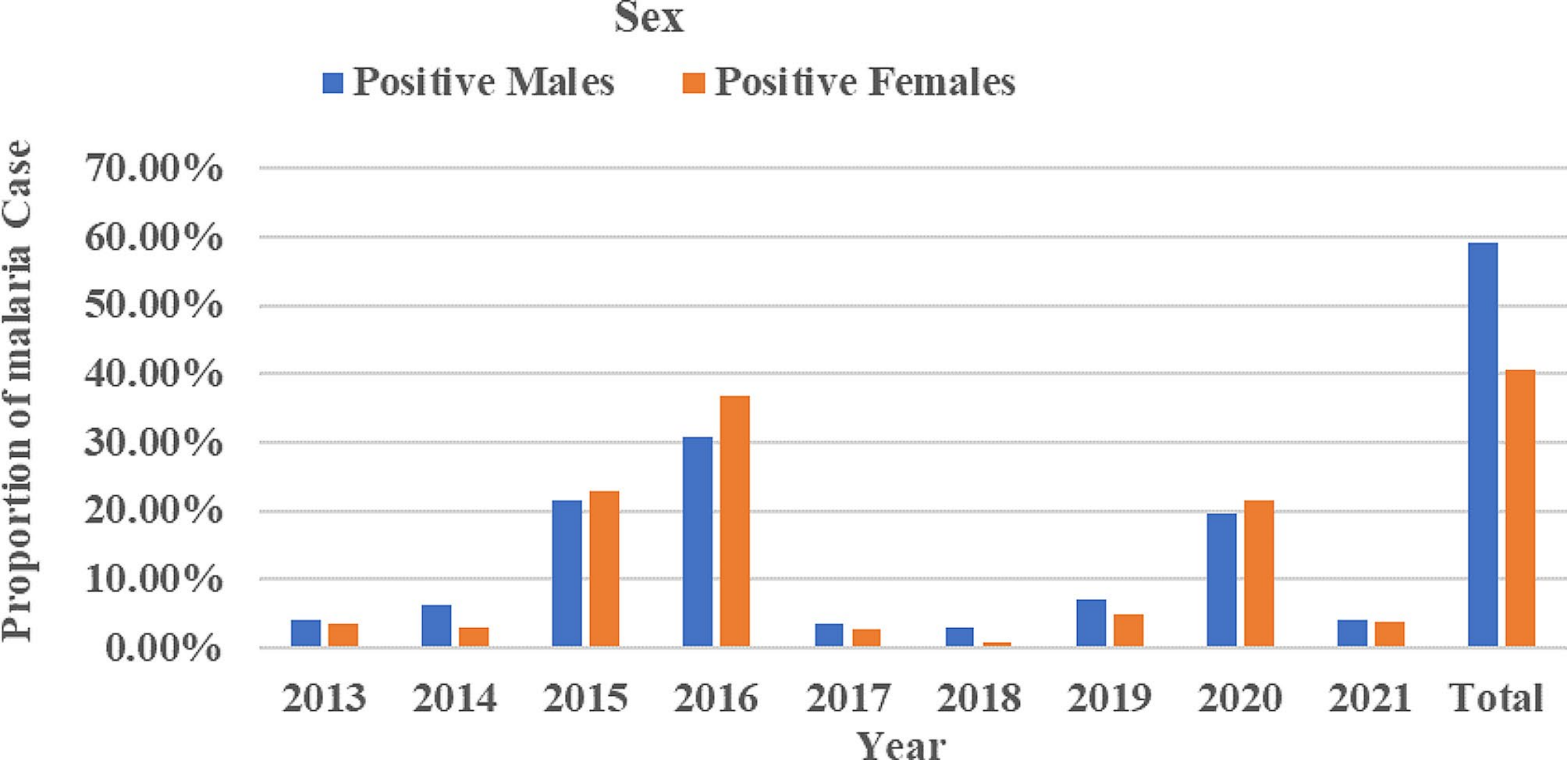
Distribution of confirmed malaria cases by sex in Addis Zemen hospital and health center, from 2013–2021

Figure 2 illustrates the total number of confirmed malaria cases by gender in the last nine years. In all confirmed malaria cases, 59.3% and 40.7% were males and females, respectively. Statistical data indicate that the overall proportion of positive cases among men was significantly higher than that of confirmed malaria cases among women (χ2 = 295.527, d.f. = 8, *p* < 0.0001). In both sexes, the lowest and highest proportion of cases occurred in 2018 and 2016, respectively (Fig. 2).

### Distribution of malaria cases by age groups

**Fig. 3:**
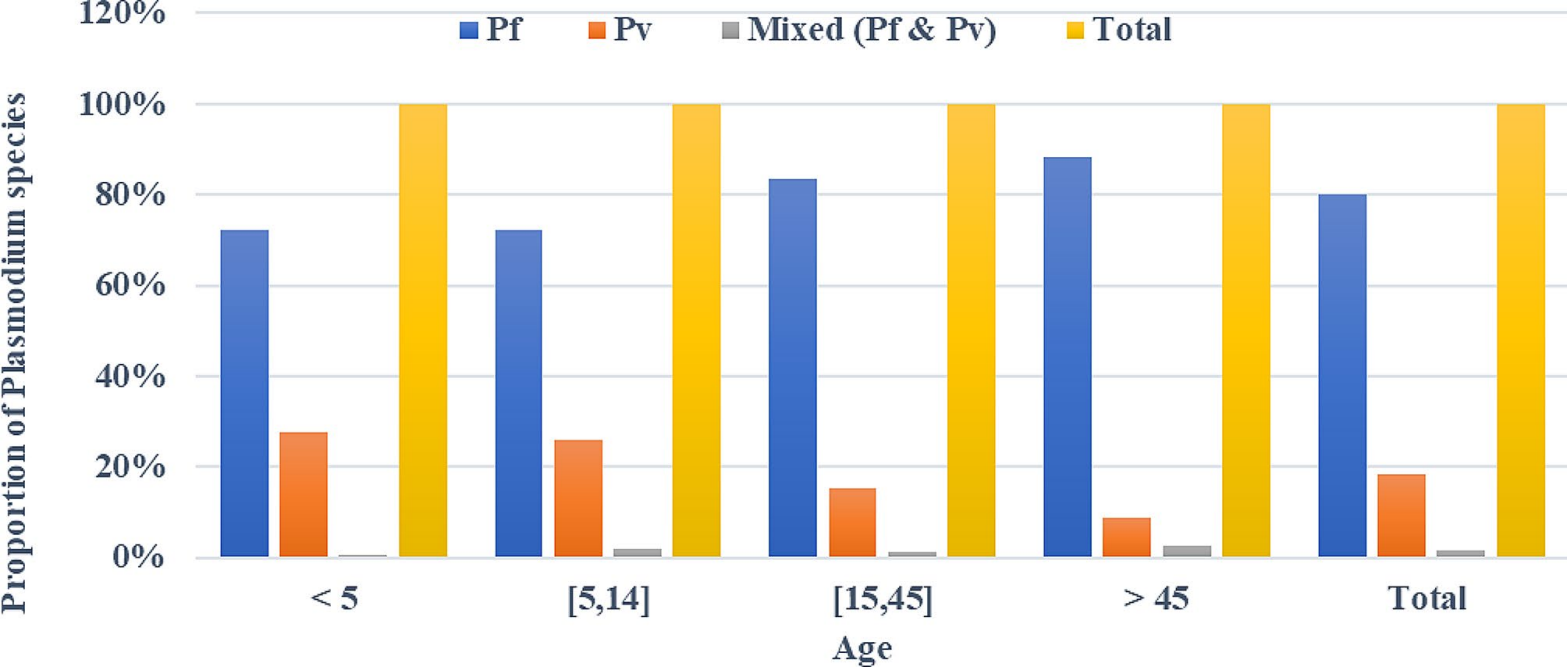
Distribution of malaria cases *(Plasmodium* species) by age in Addis Zemen hospital and health center, from 2013–2021 (Abbreviation*s’ = Plasmodium falciparum; P.v =Plasmodium vivax*; Mixed *= P.f + P.v*)

The distribution of malaria cases in relation to age is shown in Fig. 3. In all survey periods, all age groups were infected by malaria parasites; the majority of the cases were between 15 and 45 years (57%, *n* = 2635) as compared with age less than 5 (15.2%, 705), between 5 and 14 (18.3%, *n* = 847), and greater than 45 (9.5%, *n* = 439) years. The lowest malaria prevalence was seen people older than 45 years.

In connection with *Plasmodium* spp., both *P. falciparum* and *P. vivax* were affected in all age groups. However, *P. falciparum* was the predominant parasite in all age groups compared *P. vivax*. Particularly, a higher number of *P. falciparum* (59.3%) was observed in age groups ranging from 15 to 45 than the rest. Similarly, a higher proportion of *P. vivax* (47.3%) cases were reported when compared with other age groups (Fig. 3).

### Distribution of *Plasmodium* species

**Fig. 4:**
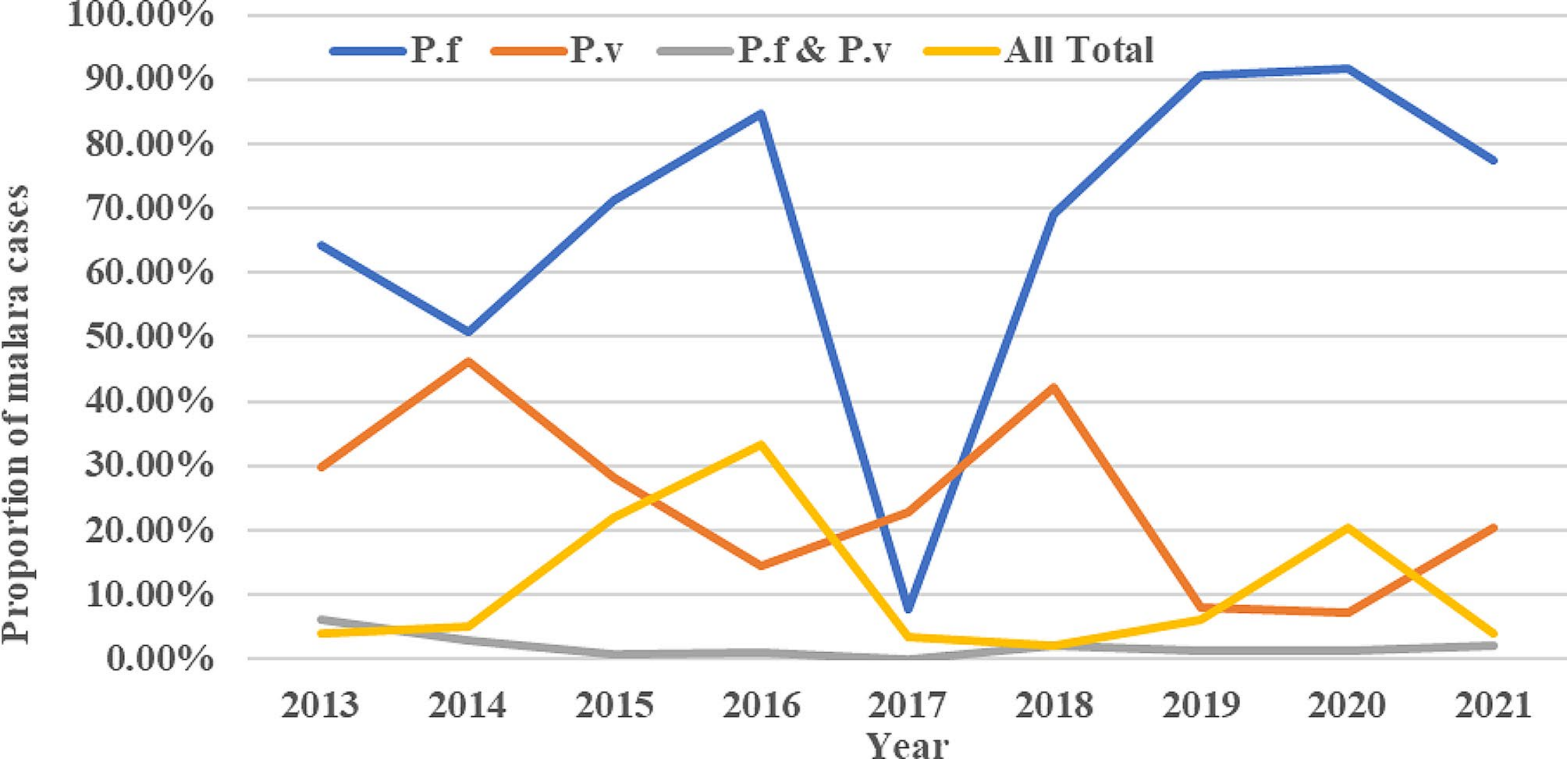
Annual trends of total malaria cases and species trends of malaria parasites in Addis Zemen hospital and health center, from 2013–2021

Throughout the reviewed periods (2013–2021), only two species of *Plasmodium* (*P. falciparum* and *P. vivax*) were found in the study area. The overall prevalence of *P. falciparum* and *P. vivax* was 80.1% (3705/4526) and 18.5% (858/4626), respectively. Moreover, 1.4% (63/4626) of mixed infections (*P. falciparum + P. vivax*) were observed. If compared the average 9-year proportion, *P. falciparum* was fourfold and fifty-eightfold more dominant than *P. vivax* and mixed infection, respectively (Fig. 4).

Statistically, a greater proportion of *P. falciparum* was recorded than *P. vivax* (χ2 = 330.079, d.f. = 8, *p <* 0.0001) in every surveyed year. In both sexes, the lowest and highest proportion of cases occurred in 2018 and 2016, respectively. The prevalence of *P. falciparum* slightly increased from 2013 and reached its peak level in 2016, then declined and reached the lowest level in 2018 (1.6%). Again, it showed an alarmingly high increment up to 2020 (23.3%). The prevalence of *P. vivax* also showed a similar tendency (Fig. 4).

### Seasonal and monthly variation of malaria cases

**Fig. 5:**
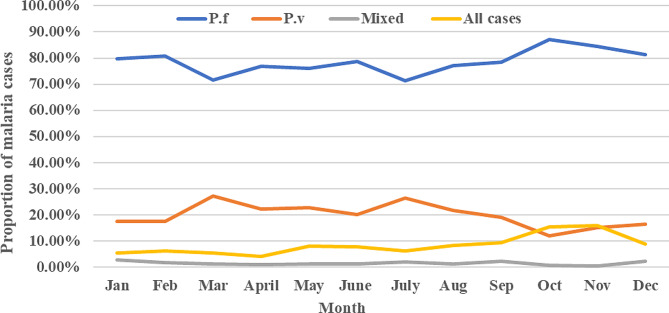
Nine-year period of malaria trends by month in Addis Zemen hospital and health center, from 2013–2021

Malaria cases were occurring throughout each month (Fig. 5), and there was a statistically significant average monthly variation (χ2 = 60.904, d.f. = 11, *p* < 0.0001). The mean monthly case was 385.5 (range 195–728). The relative highest peaks of total malaria cases were observed during the months of September, October, and November, and the lowest peaks were observed during the months of March and April. In particular, the highest *P. falciparum* cases were recorded in October and November, and the minimum cases were recorded in April and then March. Likewise, highest *P. vivax* cases were documented during the month of November, October, and September, and the fewest cases were observed in the months of April, January, and February (Fig. 5).

**Fig. 6:**
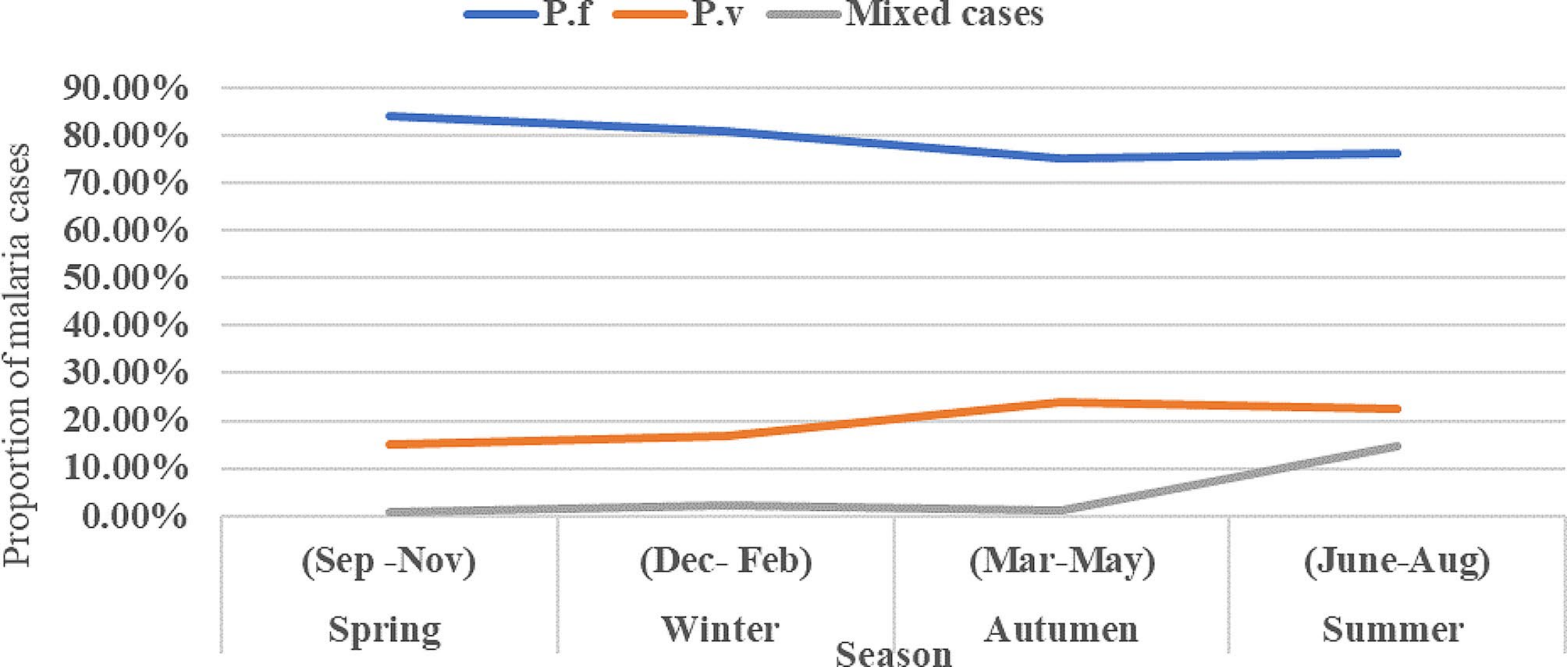
Seasonal variation of malaria transmission in Addis Zemen hospital and health center, from 2013–2021

The seasonal distribution of malaria cases is presented in Fig. 6. The prevalence of malaria occurred in all seasons across the nine years. The highest and lowest cases of malaria were observed during spring (40.20%, 1859/4626) and autumn (17.40%, 807/4626), respectively. In all seasons, the proportion of *P. falciparum* was very high in comparison with *P. vivax* (Fig. 6). In particular, more cases of *P. falciparum* and *P. vivax* were detected in the spring, and summer; while the least number was found during autumn and winter, respectively.

## Discussions

### Annual malaria case trends

Malaria is one of the most important vector-borne diseases that causes morbidity and mortality throughout the world [8]. Worldwide cases of acute illness due to malaria are estimated to be 300–500 million; however, much of the burden is laid on African children under five years of age [27] and pregnant women [1, 27]; even the health problem is worsening in SSA countries. In Ethiopia, malaria is the leading health problem due to three-fourths of the total area of the country is malaria, and more than two-thirds of the total population lives below 2,000 m of altitude [10, 11].

Potentially, this study district is one of the malaria hotspots/ hubs in Ethiopia [24, 25]. In the present study, the overall slide-positive rate of malaria was 4,626 (10.4%) in nine-year period. This result was lower as compared with other reports such as 39.6% [28], 48% [29], 33.8% [30], 21.8% [31], and 21.8% [32] done in various parts of Ethiopia. However, it was higher than 7.15% [14], 8.4% [33], 5.4% [34], and 5% [35], which were reported elsewhere in Ethiopia. This difference could be due to the presence of studies time variation, variations in geographical locations and climate, differences in population awareness about malaria bed net application, the skill of the laboratory personnel to detect and identify malaria parasites, differences in insecticide application in the districts, expansion of irrigation, insecticide and drug resistance, and differences in health-seeking behavior of suspected individuals [26, 31, 36, 37].

The Ethiopian Ministry of Health gave great attention to achieving zero indigenous malaria in districts with annual parasite incidence less than 10 and preventing the reintroduction of malaria in districts reporting zero indigenous malaria cases by 2025 via ensuring early diagnosis and prompt treatment, strengthening vector control, improving malaria surveillance and response systems, etc. [5]. However, in the current study, malaria cases were recorded throughout the year, though fluctuation was a characteristic. The highest peaks of total malaria cases were observed in 2016 (33.2%), and the lowest peaks were observed in 2018 (2%). This result agreed with a study conducted at a Bichena primary hospital, higher and lower malaria cases were recorded in similar years [38]. Similarly, a 7-year retrospective study conducted in Dembia district by Addisu et al. [31]. indicated that the highest prevalence was observed in 2016 (30.2%), followed by 2015 (24%). However, it contradicts the Ethiopian 2011 malaria indicator survey, which reported a malaria prevalence of 1.3%. This makes our finding very higher than the 2011 Ethiopian malaria indicator report [39]. This contradiction might be associated with the employed sample size, the difference in malaria control and prevention activities implemented by stakeholders, climatic factors (rainfall and temperature), the expansion of irrigation, insecticides and drug resistance. Moreover, in this study, the second peak malaria case was seen in 2020 (20.4%). The rebound-back of malaria cases this year, probably linked to the recent impact of COVID-19 on malaria control and elimination efforts. The spreading of disease across the country was collapsing the country’s malaria control and prevention strategy [5]. Consistent with this, Eshetu et al. [18]. reported the second peak malaria case in the year 2020 in Maksegnit health center, central Gondar zone, Ethiopia.

### Malaria incidence rate by sexes

During this survey, males were more affected by malaria parasites than females (*p* < 0.0001). This finding is in line with many studies conducted in different parts of Ethiopia [14, 18, 29, 31, 35, 38, 40], India [41, 42], and South Africa [43].

The presence of more infectious males than females in the present study is probably related to male work experiences and lifestyle (e.g., day labor). Males were often engaged in early night outdoor agricultural activities because irrigation activity is common in Addis Zemen. Moreover, males usually sleep outdoors to look after farms. Therefore, all these practices made males have a higher chance of exposure to be infected by the anopheles malaria vector as compared to female counterparts, who are mostly at home for taking care of children and homework purposes and protected from such infective bites. Evidence showed that much greater mosquito human-biting activities occurred outdoors than indoors during the early parts of the night, suggesting higher outdoor malaria transmission potential in Ethiopia [44]. To the reverse of our finding, a higher proportion of malaria-positive females were reported than males in Ethiopia [34] and Mozambique [45]. This variation could be coupled with the presence of low immune status of the females compared with males [46]. The presence of female house-tasks/ responsibilities/ or cooking the evening meal outdoors or reaching childbearing age or waking up before sunrise to fetch water may also put them at great risk of malaria infection [47, 48].

### Distribution of malaria cases by age groups

Regarding age, all groups were found to be infected with the malaria parasite; however, the majority of the cases were between 15 and 45 years (57%) followed by between 5 and 14 (18.3%). This was in agreement with a study conducted in Kola Diba [28], Kombolcha [14], Wolkite Health Center [49], Bichena Health Center [38], and Maksegnit Health Center [18]. However, in contrast to this finding, the study conducted in Wolita [50] and Metema Hospital [51] showed high malaria positivity in 5–14-years older than the rest age groups. The presence of more malaria infected between the 15–45 age groups in Addis Zemen town is probably linked to the regular practice of irrigation activity in the town and its surroundings [52–54]. All these practices are more exposed to productive age groups and males to anopheles mosquito bites, which can transmit *Plasmodium* parasites.

### Distribution of *Plasmodium* species

This study revealed the prevalence of *P. falciparum* (80.1%), *P. vivax* (18.5%), and mixed infections (1.4%). The existence of these two parasites is comparable with the previous documents made in Ethiopia, which were the most common parasites across the country [28, 29, 31, 38, 55]. *P. falciparum* was the most predominant species than *P. vivax*, which it exceeded about fourfold. This finding was in agreement with other previous studies conducted in Metema (*P. falciparum*, 90.7% and *P. vivax*, 9%) [51], Kola Diba (*P. falciparum*, 75% and *P. vivax*, 25%) [56], Woreta town (*P. falciparum*, 69.7% and *P. vivax*, 26.5%) [29], and selected Amhara region (Central, North and West Gondar zones) (*P. falciparum*, 73.4% and *P. vivax*, 26.6%) [55]. A previous report from WHO [57] supported this reality too, *P. falciparum* and *P. vivax* accounted for 70% and 30% of all laboratory-confirmed cases, respectively in Ethiopia. The exclusive predominance of *P. falciparum* over *P. vivax* could be that the *P. falciparum* parasite can multiply rapidly by involving more than one parasite in a single red blood cell, colonizing all ages of the red blood cells without any selection, parasite-infected red blood cells can accumulate in various organs, and the availability of *P. falciparum*-infected cases in communities [58, 59]. Furthermore, the drug resistance nature [60–62], misdiagnosed and inappropriate therapy [63], and gaps of programme performance could be the other possible reasons for such dominance.

Contrary to the current study, many other studies reported a higher proportion of *P. vivax*, than *P. falciparum* in many parts of Ethiopia [64–69]. Additionally, in Butajira *P. vivax* was 62.5% [70], in Hallaba health center *P. vivax* was 70.41% [71], in Adim Tullu district *P. vivax* was 84.6% [72], and in Wolkite health center *P. vivax* was 69.7% [49], Ethiopia. The possible explanation for this trend shifts from *P. falciparum* to *P. vivax* might be due to the public health importance of *P. vivax*, i.e., frequently overlooked and left in the shadow of the enormous problems (headache, shivering, appetite loss, anemia, nausea, vomiting) caused by *P. falciparum* [63]. In addition, the prevention and control activities of malaria as guided by the national strategic plan (2006–2010) mainly focus on *P. falciparum* because it is assumed to be more prevalent and fatal malaria in the country Ethiopia [73]. Another possible reason might be climate variability [65].

In our study, the proportion of both *P. falciparum* and *P. vivax* showed a slight increment from 2013 and reached a peak level in 2016, and then declined and reached the lowest level in 2018. Again, they showed an alarming rise in 2020. Consistent with this finding, Minwuyelet & Aschale [38] reported high and low prevalence levels of *P. falciparum* and *P. vivax* in Bichena primary hospital in 2016 (6.81%, 2.67%) and 2018 (2.13%, 1.65%), respectively. On the contrary, other retrospective studies [16, 32, 55, 74, 75] done in various parts of Ethiopia indicated that there was an overall decline in malaria incidence associated with a decrement of *P. falciparum* and *P. vivax*. The lack of smooth reduction in the present study could be connected with COVID-19 [5] and the absence of better practices of malaria prevention and control strategies, i.e., the use of LLINs alone, IRS alone, or the use of improper insecticide-treated bed nets [76]. Besides, in Addis Zemen town irrigation is common and farmers may spend most of their time in the field, which exposes them to mosquito bites [77].

### Seasonal and monthly variation of malaria cases

Malaria cases were occurring throughout the months of each year and showed significant variation (χ2 = 60.904, d.f.=11, *p* < 0.0001). The highest peaks of total malaria cases were observed during September, October, and November, and the lowest peaks were observed during March and April. In particular, the highest *P. falciparum* cases were recorded in September, October, and November and the minimum cases were recorded in April and then March. Likewise, the highest *P. vivax* cases were documented during September, November, October, and the least cases were observed in April, January, and February. Our findings are consistent with other studies conducted in Bichena Primary Hospital [38], Woreta Health Center [34], and Boricha district [32]. Moreover, other previously made studies [14–16] in various parts of Ethiopia also documented the presence of increased total malaria cases in September, October, and November. These months are considered the peak malaria transmission period in Ethiopia after the heavy rain in July and August [5, 78].

Regarding the seasonal distribution of malaria, in our findings, the highest and the lowest cases of malaria were observed during spring (40.20%) and autumn (17.40%), respectively. In line with this, Getacher et al. [15], Alkadir et al. [16], and Minwuyelet & Aschale [38] reported highest and lowest peak of total malaria cases in spring and autumn in Bichena primary hospital and Ataye district, Ethiopia, respectively. Moreover, Alemu et al. [65]. in Jimma town, Gemechu et al. [79]. in Sibu Sire district, Alelign et al. [29]. in Woreta town, and Gebretsadik et al. [14]. in Kombolcha health center, Ethiopia, had reported peak malaria cases in spring than in other seasons. This season is preferable for mosquito breeding because it provides appropriate temperature and enough rainfall for them. In Ethiopia, the peak malaria transmission occurs between September and December following the June to September long rains [73, 78]. Different from this result, Eshetu et al. [18]. reported highest malaria cases in Autumn than in other seasons in Maksegnit Health Center. The review of Alelign et al. [29]. also indicated that Autumn was the second peak malaria season in Woreta town. This would be an important indication that the area needs due attention and further concerted malaria interventions.

## Conclusions

The study indicated that malaria remains one of the most important causes of morbidity in our study area with a high slide positivity rate (10.4%). Malaria cases were occurring in all months and throughout the nine years, but the malaria transmission was peaking from September to November, coinciding with the major malaria transmission season in Ethiopia. In addition, cases of malaria were more frequent in men than in women, and the age group between 15 and 45 was very affected, which represents the productive segment of the Ethiopian population [80]. Two of the most important *Plasmodium* species were identified, *P. falciparum* and *P. vivax*; however, *P. falciparum* is the most predominant. These species were found in all the reviewed months and years in the study area. This could be a great challenge to the success of the ongoing malaria elimination program in Ethiopia. In this study, malaria transmission remains high, especially in the spring season. Hence, proper season-based LLINs (duration and timing of warm and wet climate) use and regular supervision of the ongoing irrigation activity, pocket water management due to Sheni River water reduction, health education, and immediate patient treatment are needed to alleviate the problem.

## Data Availability

All relevant data are available on the manuscript. If necessary and reasonably requested, all used data in the manuscript are available in the hand of corresponding author.

## Abbreviations

WHO: World Health Organization
SSA: sub-Saharan African
MoH: Ministry of Health
LLINs: Long Lasting Insecticide Treated Nets
IRSs: Indoor Residual Sprayings
m.a.s.l.: meter above sea level
SPSS: Statistical Package for the Social Science
